# *LBD16* and *LBD18* acting downstream of *ARF7* and *ARF19* are involved in adventitious root formation in Arabidopsis

**DOI:** 10.1186/s12870-019-1659-4

**Published:** 2019-01-31

**Authors:** Han Woo Lee, Chuloh Cho, Shashank K. Pandey, Yoona Park, Min-Jung Kim, Jungmook Kim

**Affiliations:** 1 Department of Bioenergy Science and Technology, Chonnam National University, Yongbongro 77, Buk-gu, Gwangju, 61186 South Korea; 2 Kumho Life Science Laboratory, Chonnam National University, Gwangju, 61186 South Korea

**Keywords:** *Arabidopsis thaliana*, Adventitious root formation, Auxin response factor, Lateral organ boundaries domain, *LBD16*, *LBD18*

## Abstract

**Background:**

Adventitious root (AR) formation is a complex genetic trait, which is controlled by various endogenous and environmental cues. Auxin is known to play a central role in AR formation; however, the mechanisms underlying this role are not well understood.

**Results:**

In this study, we showed that a previously identified auxin signaling module, *AUXIN RESPONSE FACTOR(ARF)7/ARF19-LATERAL ORGAN BOUNDARIES DOMAIN(LBD)16/LBD18* via AUXIN1(AUX1)/LIKE-AUXIN3 (LAX3) auxin influx carriers, which plays important roles in lateral root formation, is involved in AR formation in Arabidopsis. In *aux1*, *lax3*, *arf7*, *arf19*, *lbd16* and *lbd18* single mutants, we observed reduced numbers of ARs than in the wild type. Double and triple mutants exhibited an additional decrease in AR numbers compared with the corresponding single or double mutants, respectively, and the *aux1 lax3 lbd16 lbd18* quadruple mutant was devoid of ARs. Expression of *LBD16* or *LBD18* under their own promoters in *lbd16* or *lbd18* mutants rescued the reduced number of ARs to wild-type levels. LBD16 or LBD18 fused to a dominant SRDX repressor suppressed promoter activity of the cell cycle gene, *Cyclin-Dependent Kinase(CDK)A1;1*, to some extent. Expression of *LBD16* or *LBD18* was significantly reduced in *arf7* and *arf19* mutants during AR formation in a light-dependent manner, but not in *arf6* and *arf8*. GUS expression analysis of promoter-GUS reporter transgenic lines revealed overlapping expression patterns for *LBD16*, *LBD18*, *ARF7*, *ARF19* and *LAX3* in AR primordia.

**Conclusion:**

These results suggest that the *ARF7/ARF19*-*LBD16/LBD18* transcriptional module via the AUX1/LAX3 auxin influx carriers plays an important role in AR formation in Arabidopsis.

**Electronic supplementary material:**

The online version of this article (10.1186/s12870-019-1659-4) contains supplementary material, which is available to authorized users.

## Background

Root architecture in higher plants, which is critical for anchorage in soil and the uptake of water and nutrients, is diverse at both the system and anatomical levels [1]. In general, dicotyledonous plants, such as *Arabidopsis thaliana*, have a primary root that branches to form lateral roots (LRs). In both monocot and dicotyledonous plants, the primary root can develop adventitious roots (ARs) that arise naturally from the aerial organs as an adaptive response to environmental changes, such as flooding and dark-light transitions, or artificially by wounding [2–5]. AR formation is critical for vegetative propagation of elite genotypes in agriculture and is also important for plant survival under a variety of biotic and abiotic stresses [4, 5]. Auxin plays a central role in both LR and AR formation [6]. Although signaling and molecular mechanisms of auxin-regulated primary and LR development are relatively well characterized, our understanding of how auxin regulates AR formation is rudimentary [1, 7–9].

Plant root development is regulated by establishing auxin maxima at the primordium tip through auxin transport [10–13]. During auxin transport, auxin travels acropetally or basipetally over long distances by the combined action of plasma membrane-localized auxin efflux and influx carriers and triggers various regulatory mechanisms along its path [14–17]. Auxin efflux carriers, including two major transmembrane proteins, i.e., PIN-FORMED (PIN) and ATP-binding cassette subfamily B (ABCB), were shown to be involved in both LR and AR formation in different plant species [10, 18–22]. Studies with the polar auxin transport inhibitor, *N*-1-naphthylphthalamic acid, showed that PIN1-mediated auxin transport is necessary for AR emergence in rice plants [23]. Auxin together with ethylene positively regulates AR initiation through *DIAGEOTROPICA* (*DGT*), which encodes a cyclophilin A-type protein (SlCYP) [24, 25]. SlCYP1 changes the abundance of PIN efflux carriers at the plasma membrane to modulate polar auxin transport during AR initiation [26–29].

Auxin signaling is regulated by two large protein families: the AUXIN RESPONSE FACTOR (ARF) proteins, which act as the DNA-binding transcriptional regulators of auxin responses, and the Aux/IAA proteins, negative regulators of ARFs [30]. Several *ARF*s have been identified as playing a role in AR formation in both Arabidopsis and rice. *ARF6* and *ARF8* have been shown to act as positive regulators of AR initiation in Arabidopsis hypocotyls, whereas *ARF17* acts as a negative regulator [31, 32]. These *ARF*s regulate each other’s expression at the transcriptional and posttranscriptional levels by modulating the homeostasis of *miR160*, which targets *ARF17* and *miR167*, subsequently targeting both *ARF6* and *ARF8* [32]. This complex network of transcription factors regulates the expression of three auxin-inducible *GRETCHEN HAGEN3* (*GH3*) genes, encoding acyl-acid-amido synthetases, which are required for fine-tuning AR initiation in the Arabidopsis hypocotyls by modulating jasmonic acid homeostasis [33]. Some studies have indicated that auxin signaling modules involved in LR formation could play a role in AR formation as well. For instance, *ARF7* and *ARF19* control LR formation as well as AR formation in Arabidopsis [34–38]. *OsARF16*, which is a rice ortholog of *ARF7* and *ARF19*, controls the initiation of adventitious crown root primordia in rice by activating the expression of *CROWN ROOTLESS1/ADVENTITIOUS ROOTLESS1* (*CRL1/ARL1*), which encodes a LATERAL ORGAN BOUNDARIES DOMAIN (LBD) protein [39–41].

In Arabidopsis, several *LBD* genes, such as *LBD16*, − *18*, − *29* and − *33*, have been demonstrated to play critical and distinct roles in auxin-regulated LR development [37, 42–49]. It has been shown that the auxin influx carriers *AUXIN1* (*AUX1*) and *LIKE-AUX1 3* (*LAX3*) are required for auxin-responsive expression of *LBD16* and *LBD18* to control various stages of LR development in Arabidopsis [45, 50, 51]. In the present study, we show that the *AUX1/LAX3*-*ARF7/ARF19*-*LBD16/LBD18* signaling module is also important for AR formation in Arabidopsis, providing evidence of a common regulatory mechanism being utilized for LR and AR formation during auxin signaling.

## Results

### Analysis of GUS expression patterns of *Pro*_*ARF7*_*:GUS*, *Pro*_*ARF19*_*:GUS*, *Pro*_*LAX3*_*:GUS*, *Pro*_*LBD16*_*:GUS* and *Pro*_*LBD18*_*:GUS* during AR development

**Fig. 1:**
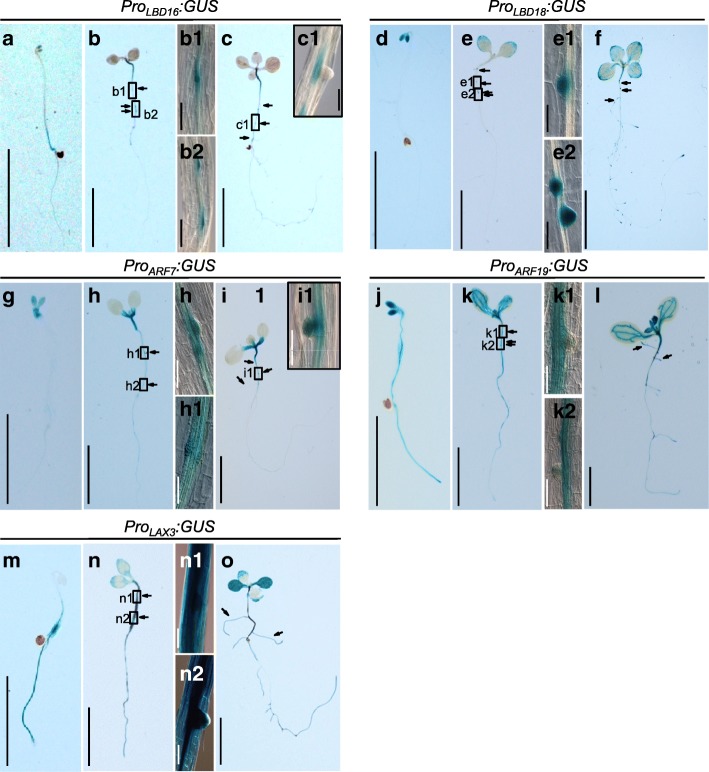
GUS expression in hypocotyls of *Pro*_*LBD16:*_*GUS, Pro*_*LBD18*_*:GUS, Pro*_*ARF7:*_*GUS, Pro*_*ARF19:*_*GUS* and *Pro*_*LAX3*_*:GUS* transgenic plants. **a-c** GUS staining for the expression of *Pro*_*LBD16*_*:GUS,* **d-f** *Pro*_*LBD18*_*:GUS,* **g-i** *Pro*_*ARF7:*_*GUS,* **j-l** *Pro*_*ARF19:*_*GUS* and **m-o** *Pro*_*LAX3*_*:GUS* in seedlings grown in the dark for 3 d (a, d, g, j and m) and then in the light for 72 h (b, e, h, k and n) or 6 d (c, f, i, l and o). Magnified images of the regions boxed in b, c, e, h, i, k and n are shown in b1, b2, c1, e1, e2, h1, h2, i1, k1, k2, n1 and n2. Arrows point to ARs or primordia. Bars = 1 cm in a-o and 50 μm in b1, b2, c1, e1, e2, h1, h2, i1, k1, k2, n1 and n2

To gain insights into the function of the *LAX3-ARF7/ARF19-LBD16/LBD18* signaling module during AR development, we analyzed GUS expression in *Pro*_*LBD16*_*:GUS*, *Pro*_*LBD18*_*:GUS*, *Pro*_*ARF7*_*:GUS*, *Pro*_*ARF19*_*:GUS* and *Pro*_*LAX3*_*:GUS* transgenic plants during the early stages of AR formation (Fig. 1). GUS expression was detected in the cotyledon and lower part of the hypocotyl of 3-d-old dark-grown *Pro*_*LBD16*_*:GUS* seedlings at time T0 (Fig. 1a). After transferring these seedlings to the light for 72 h, GUS expression was clearly detected in the early AR primordium in the hypocotyl (Fig. 1b). After 6 d in the light, GUS expression generally increased in both the hypocotyl and root and was detected in the hypocotyl stele tissue near the emerged AR (Fig. 1c). Regarding *Pro*_*LBD18*_*:GUS*, GUS expression was detected only in the hypocotyl of 3-d-old dark-grown seedlings (Fig. 1d): after transferring to the light for 72 h, strong GUS expression was detected in the AR primordium in the hypocotyl (Fig. 1e). In *Pro*_*ARF7*_*:GUS*, *Pro*_*ARF19*_*:GUS* and *Pro*_*LAX3*_*:GUS* seedlings, GUS expression was detected in both the hypocotyl stele tissue and AR primordium after transferring 3-d-old dark-grown seedlings to the light for 72 h (Fig. 1g–o). These overlapping and distinctive GUS expression patterns in the hypocotyl stele tissue and AR primordium of the GUS reporter transgenic lines indicated that *LBD16* and *LBD18* may play an overlapping role in early AR primordium development and *LBD18* may play a distinctive role in the AR primordium in later developmental stages downstream of *ARF7/ARF19-LAX3* during AR development.

### *AUX1*, *LAX3*, *LBD16* and *LBD18* are involved in AR formation in Arabidopsis hypocotyls

**Fig. 2:**
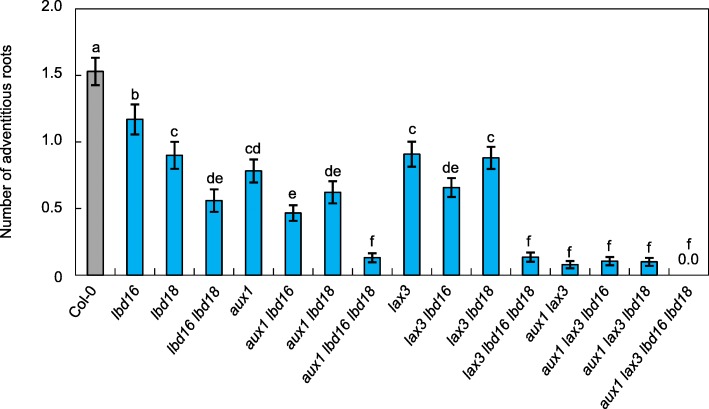
Number of ARs in the hypocotyls of wild-type (Col-0), *lbd16*, *lbd18*, *aux1* and *lax3* single and multiple mutants. ARs were measured in seedlings that were grown on vertical plates for 3 d in darkness, until their hypocotyls were 6 mm long, and then transferred to the light for 7 d. Data are presented as means ± SE, determined from at least 90 seedlings. Different letters indicate a significant difference determined by one-way ANOVA followed by Duncan’s post hoc test (*P* < 0.05)

**Fig. 3:**
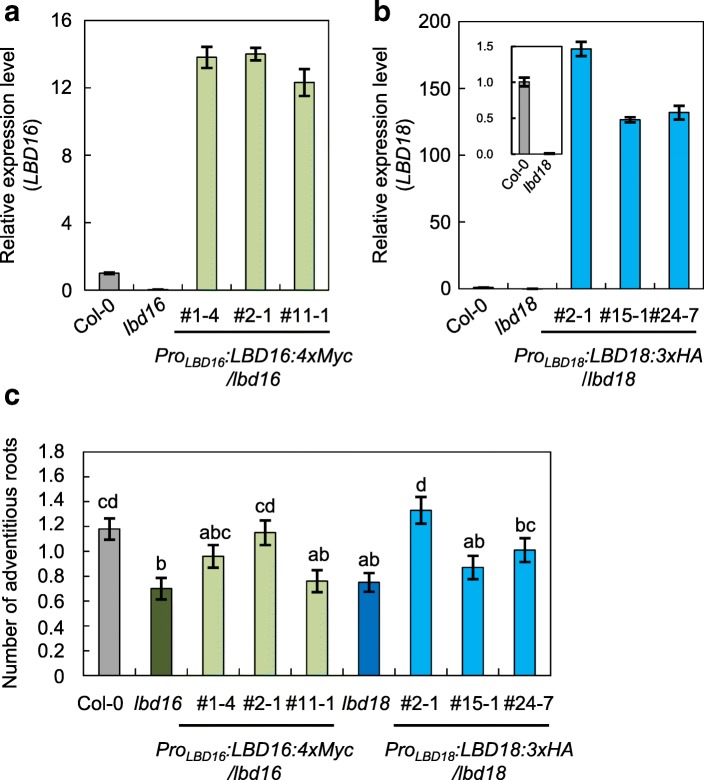
Complementation analysis of AR formation of *lbd16* and *lbd18*. **a** Expression analysis of *LBD16* in Col-0, *lbd16* and *Pro*_*LBD16*_*:LBD16:Myc/lbd16* plants*.* **b** Expression analysis of *LBD18* in Col-0, *lbd18* and *Pro*_*LBD18*_*:LBD18:3XHA*/*lbd18* plants*.* Seven-d-old seedlings were used for real-time RT-PCR analysis (A and B). The relative fold changes were plotted after normalization to *ACTIN7*. The relative fold change represents the ratio in the mutant or transgenic plants relative to the transcript level in Col-0 plants. Mean ± SE values were determined from three technical replicates. **c** Number of ARs of *Pro*_*LBD16*_*:LBD16:myc/lbd16* and *Pro*_*LBD18*_*:LBD18:HA/lbd18* plants*.* Numbers above the lines indicate the line numbers of transgenic plants. The number of adventitious roots was analyzed as described in Fig. 2. Data are presented as means ± SE, determined from 100 seedlings. Different letters indicate a significant difference determined by one-way ANOVA followed by Duncan’s post hoc test (*P* < 0.05)

To determine the roles of auxin influx carriers, AUX1 and LAX3, and two critical LBD transcription factors, LBD16 and LBD18, in AR formation, we measured AR numbers on hypocotyls from single and multiple mutants derived from *aux1*, *lax3*, *lbd16* and *lbd18* (Fig. 2). To induce AR formation in hypocotyls, seeds germinated were etiolated vertically for 3 d and grown in the light for 7 d, and the ARs were counted [32]. The *aux1*, *lax3*, *lbd16* and *lbd18* single mutants showed a significant decrease in AR numbers compared with that of the wild type after transferring 3-d-old dark-grown seedlings to the light for 7 d (49.01, 41.17, 23.52 and 41.17% for *aux1*, *lax3*, *lbd16* and *lbd18,* respectively) (Fig. 2). The number of ARs in double mutants, *lbd16 lbd18*, *aux1 lbd16*, *aux1 lbd18*, *lax3 lbd16*, *lax3 lbd18* and *aux1 lax3*, was further reduced compared with that of their corresponding single mutants (Fig. 2). AR numbers in triple mutants, *aux1 lbd16 lbd18* and *lax3 lbd16 lbd18,* were further reduced to ~ 8% compared to that of the wild type, whereas the *aux1 lax3* double mutant and *aux1 lax3 lbd16* and *aux1 lax3 lbd18* triple mutants exhibited 3.5–4% that of the wild-type AR number (Fig. 2). In the quadruple mutant, *aux1 lax3 lbd16 lbd18*, AR formation could not be detected. These results indicate that auxin influx carriers, such as AUX1 and LAX3, are critical for AR formation and that *AUX1*, *LAX3*, *LBD16* and *LBD18* genes are essential for AR formation. To further confirm the role of *LBD16* and *LBD18* in AR formation, we generated transgenic Arabidopsis plants expressing *LBD16:MYC* (*Pro*_*LBD16*_*:LBD16:Myc/lbd16*) or *LBD18:HA* (*Pro*_*LBD18*_*:LBD18:HA/lbd18*) in *lbd16* or *lbd18* mutant background under the control of their own promoter, respectively, (Fig. 3a, b), and analyzed AR numbers of these complementation lines. The three different complementation lines selected for AR analysis rescued AR development defects caused by *lbd16* or *lbd18* mutations to varying degrees (Fig. 3c). *Pro*_*LBD16*_*:LBD16:Myc/lbd16* (#2–1) and *Pro*_*LBD18*_*:LBD18:HA/lbd18* (#2–1) transgenic mutant lines showed wild-type levels of AR numbers (Fig. 3c). These results demonstrated that *LBD16* and *LBD18* play significant roles in AR development.

### Both LBD16:SRDX and LBD18:SRDX suppress cell cycle gene promoter activities during induction of AR formation

**Fig. 4:**
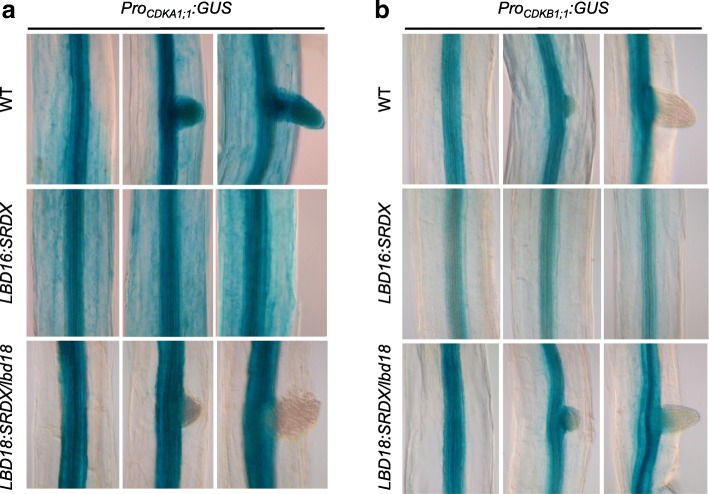
GUS expression during AR development in Arabidopsis plants harboring the promoter of cell cycle genes fused to *GUS* reporter and *Pro*_*LBD16*_*:LBD16:SRDX* (**a**) or *Pro*_*LBD18*_*:LBD18:SRDX* (**b**) in *lbd18* mutants

Previous studies have shown that *LBD16* and *LBD18* regulate expression of some cell cycle genes during LR initiation [45, 48]. To examine if *LBD16* and *LBD18* might be involved in the expression of cell cycle genes during AR formation, we used transgenic Arabidopsis expressing LBD16:SUPERMAN REPRESSIVE DOMAIN X (SRDX) or LBD18:SRDX under the control of their own promoters and harboring *Cyclin-Dependent Kinase (CDK)A1;1* or *CDKB1:1* in Col-0 or in *lbd18* mutants [45]. We found that LBD16:SRDX suppressed GUS expression in the vasculature of the hypocotyl in *Pro*_*CDKB1;1*_*:GUS* transgenic plants, whereas LBD18:SRDX suppressed GUS expression in the primordium and vasculature in the hypocotyl of *Pro*_*CDKA1;1*_*:GUS* transgenic plants (Fig. 4). Suppression of GUS expression in the hypocotyl vasculature of *Pro*_*CDKB1;1*_*:GUS* by LBD16:SRDX or in the AR of *Pro*_*CDKA1;1*_*:GUS* by LBD18:SRDX correlates with preferential expression of *LBD16* or *LBD18* in the hypocotyl stele tissue or only in the AR primordium, respectively. We also noted that LBD16:SRDX completely blocked AR formation in the hypocotyl in the light, whereas LBD18:SRDX did not block AR formation (Fig. 4). These results indicated that *LBD16* and *LBD18* might be involved in the expression of some cell cycle genes during AR development at distinctive stages. However, further quantitative and functional analyses are necessary to clarify the direct involvement of *LBD16* and *LBD18* in regulating cell cycle gene expression during AR development.

### *LBD16* and *LBD18* act downstream of *ARF7* and *ARF19* to regulate AR development

**Fig. 5:**
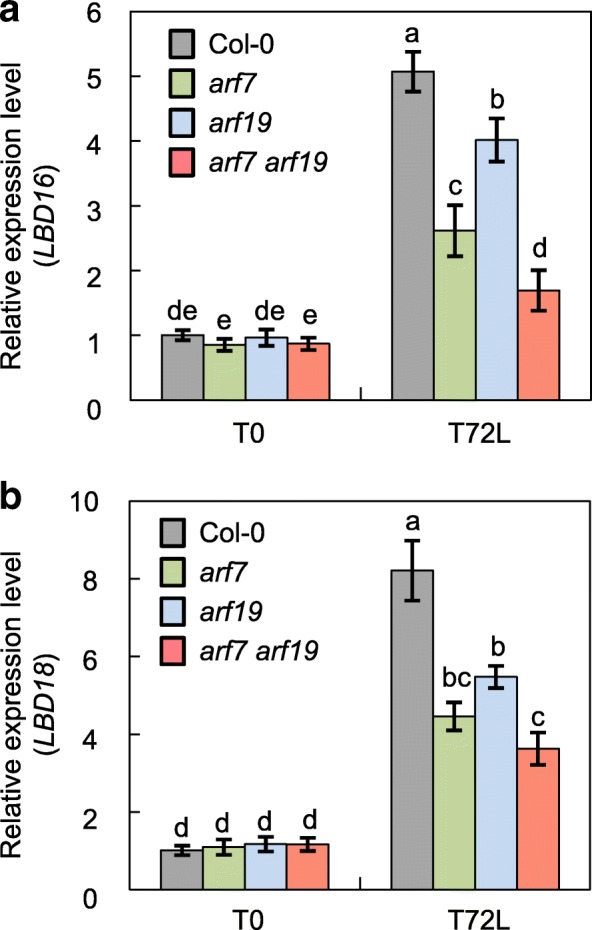
Expression analyses of *LBD16* and *LBD18* in hypocotyls of Col-0, *arf7*, *arf19* and *arf7 arf19* mutants. Expression of *LBD16* (**a**) and *LBD18* (**b**) transcripts in hypocotyls of Col-0, *arf7*, *arf19* and *arf7 arf19*. Seedlings were etiolated in the dark until their hypocotyls were 6 mm long (T0) and then transferred to the light for 72 h (T72L). The seedlings were then harvested for RT-qPCR analysis. The relative fold change represents the ratio of the transcript level in the mutants relative to the transcript level in Col-0 at T0. Mean ± SE values were determined from three biological replicates (each biological replicate was estimated as the average of two technical RT-qPCR replicates). Different letters indicate a significant difference determined by one-way ANOVA followed by Duncan’s post hoc test (*P* < 0.05)

During LR development, *LBD16* and *LBD18* are transcriptionally regulated downstream of *ARF7* and *ARF19* during auxin signaling [34, 37, 52]. We thus investigated whether the same transcriptional module functions during AR formation in a light-dependent manner. To this end, we analyzed expression of *LBD16* and *LBD18* in hypocotyls of *arf7* and *arf19* single mutants and an *arf7 arf19* double mutant at different time points during the early stages of AR formation by RT-qPCR analysis (Fig. 5). At T0, which is the etiolated stage of seedlings (just before transferring to light), the expression of *LBD16* and *LBD18* was unchanged in all three mutants compared with that of the wild type (Fig. 5). After transferring to the light for 72 h (T72L), the expression of *LBD16* and *LBD18* was enhanced in the wild type as well as in all mutant plants. However, light-induced expression of *LBD16* and *LBD18* was significantly reduced in *arf7* (by 48.5 and 45.6%*,* respectively) and *arf19* mutants (20.9 and 33.3%, respectively) compared with that of the wild type (Fig. 5a). *LBD16* and *LBD18* expression were further reduced in *arf7 arf19* double mutants (by 66.6 and 55.9%, respectively), compared with that of the *arf7* and *arf19* single mutants (Fig. 5b), indicating that *ARF7* and *ARF19* regulate *LBD16* and *LBD18* expression during AR development after the dark-light transition.

**Fig. 6:**
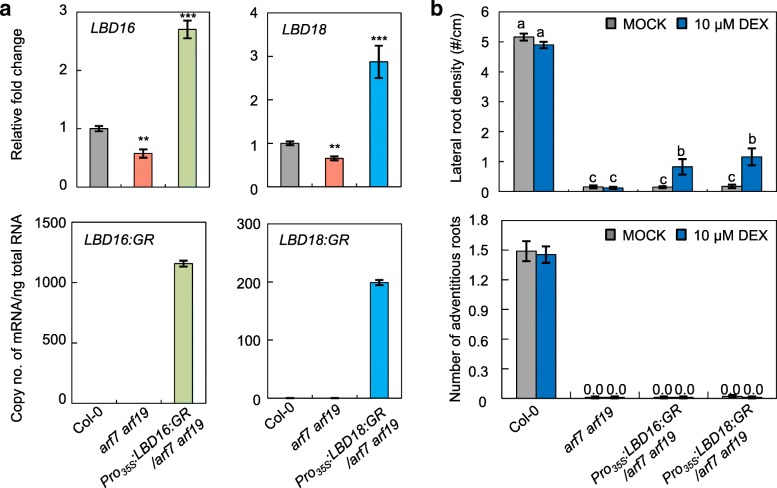
Analysis of LR and AR formation in *arf7 arf19* mutants expressing *LBD16:GR* or *LBD18:GR*. **a** Expression analysis of *LBD16* and *LBD16:GR* in Col-0, *arf7 arf19* and *Pro*_*35S*_*:LBD16:GR/arf7arf19* (left panels) and *LBD18* and *LBD18:GR* in Col-0, *arf7 arf19* and *Pro*_*35S*_*:LBD18:GR/arf7arf19* plants (right panels)*.* Seven-d-old seedlings were used for real-time RT-PCR analysis. In the left panels, the relative fold changes were plotted after normalization to *ACTIN7*. The relative fold change represents the ratio of the transcript level in the mutant relative to the transcript level in Col-0 plants. In the right panels, copies of the transcripts were plotted per ng of total RNA after normalization to *ACTIN7* RNA. Mean ± SE values were determined from three technical replicates. **b** Lateral root densities and number of ARs in the hypocotyls of Col-0, *arf7 arf19, Pro*_*35S*_*:LBD16:GR/arf7arf19* and *Pro*_*35S*_*:LBD18:GR/arf7arf19* plants. The LR densities were plotted by LR numbers (#) per unit primary root length (cm) measured from plants grown vertically for 2 weeks and shown in the top panel. Data are presented as means ± SE determined from 20 seedlings. Different letters indicate a significant difference determined by one-way ANOVA followed by Duncan’s post hoc test (*P* < 0.05). ARs were measured in seedlings that were grown on vertical plates for 3 d in darkness and then transferred to the 16-h photoperiod for 2 weeks with or without DEX and plotted in the bottom panel. Data are presented as means ± SE determined from 100 seedlings

In a previous study, overexpression of *LBD16* or *LBD18* in *arf7 arf19* mutant under the control of the *Cauliflower mosaic virus* (CaMV 35S) promoter resulted in induction of notable LR formation within 12 d, demonstrating that *LBD16* and *LBD18* can independently function downstream of *ARF7* and *ARF19* to control LR development [37, 42]. As reported in previous studies [37, 42], the LR phenotype of the *arf7 arf19* mutant was rescued by *LBD16* or *LBD18* overexpression to some extent (Fig. 6b, top panel). We next examined whether overexpression of *LBD16* or *LBD18* in *arf7 arf19* mutants could cause ectopic initiation of ARs, but could not detect any AR formation even after 2 weeks of plant growth in the light (Fig. 6b, bottom panel). This result suggested that unlike LR initiation, *LBD16* and *LBD18* require additional factors produced by ARF7 and ARF19 to induce AR initiation.

Previous studies have shown that *ARF6* and *ARF8* act as positive regulators of AR formation [31, 32]. Thus, we tested if *ARF6* and *ARF8* could control the expression of *LBD16* and *LBD18* for AR formation. The time-course response of *LBD16* and *LBD18* expression in *arf6* and *arf8* mutants after treatment with auxin indole-3-acetic acid was analyzed using RT-qPCR, but no alteration in the expression of *LBD16* or *LBD18* in *arf6* and *arf8* mutant backgrounds was observed compared with that of the wild type (Additional file 1: Figure S1), suggesting that *ARF6* and *ARF8* regulate AR formation through a distinct pathway, independent of *LBD16* and *LBD18*, during auxin signaling. Taken together, these results indicated that *LBD16* and *LBD18* expression is regulated downstream of *ARF7* and *ARF19*, but not of *ARF6* and *ARF8*, for AR development.

## Discussion

Studies on genetic aspects and hormonal responses of LR and AR formation suggested that LRs and ARs share key elements of genetic and hormonal regulatory networks but with different regulatory mechanisms [1]. While genetic components and molecular signaling pathways during LR development in Arabidopsis have been well characterized [9], those involved in AR development are largely unknown. In this work, we showed that the auxin-responsive *ARF7*/*ARF19*-*LBD16*/*LBD18* transcriptional module, via AUX1/LAX3 transporters, plays an important role in AR formation in the Arabidopsis hypocotyl.

In Arabidopsis, the developmental processes of LRs are well defined, and consists of the priming of pericycle cells in the basal meristem of the primary root, the first anticlinal asymmetric cell division (initiation), ordered anticlinal and periclinal cell divisions to form a dome-shaped LR primordium, and the emergence of an LR primordium from the primary root [53, 54]. Although the developmental stages of AR formation are not well described in Arabidopsis, AR formation in apple cuttings have been similarly divided into four successive phases: cell dedifferentiation, induction as the beginning of cell division, the outgrowth of a dome-shaped primordium, and the emergence of the AR [55]. In Arabidopsis, ARs initiate from the pericycle cells adjacent to the xylem pole in the hypocotyl, similar to how LRs initiate [56]. ARF proteins, which are involved in LR initiation, were found to regulate AR initiation in Arabidopsis [32, 33], indicating that although AR and LR originate from different organs, similar molecular mechanisms may regulate AR initiation. In rice, CRL1/ARL1, which promotes crown root initiation, positively regulates 277 genes, and among those, it positively regulates many genes homologous to Arabidopsis genes involved in LR formation [57].

**Fig. 7:**
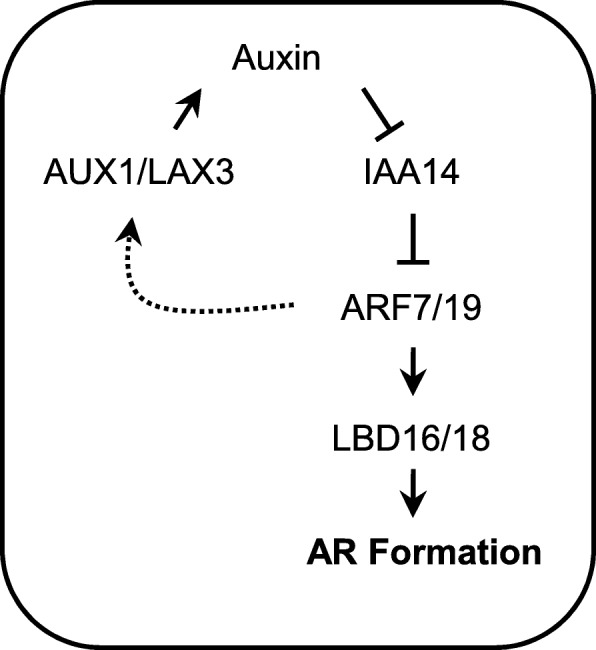
A model showing the auxin signaling module *ARF7/19-LBD16/18* via AUX1/LAX3 auxin influx carriers mediated AR formation in Arabidopsis

In the present study, we showed that the signaling module of *AUX1/LAX3-ARF7/ARF19-LBD16/LBD18*, which has been shown to regulate LR formation [37, 42, 45, 48], is involved in AR formation (Fig. 7), indicating conservation of developmental processes for AR and LR formation. However, we noted some differences in the roles of each signaling component between LR and AR development. We had previously found that quadruple mutations in *lbd16 lbd18 aux1 lax3* nearly abolished the formation of emerged LRs, but barely affected LR primordium density at early developmental stages from I to III [45]. However, we could not detect any AR primordium in this quadruple mutant (Fig. 2). This observation indicates that the LBD16/LBD18 transcriptional module downstream of AUX1/LAX3 is essential for both AR initiation and development, whereas the same signaling module functions redundantly during LR initiation possibly in conjunction with other *LBD* genes. Moreover, we found that overexpression of *LBD16* or *LBD18* in the *arf7 arf19* mutant could not rescue AR defects, whereas the same approach significantly stimulated LR formation in the *arf7 arf19* mutant (Fig. 6) [34, 37], indicating that LBD16 and LBD18 require additional components produced by ARF7 and ARF19 to regulate AR formation. In addition, LBD29 plays an important role in LR formation downstream of ARF7 by directly activating *LAX3* expression in response to auxin [47, 49]. *LBD18* expression is regulated downstream of *LAX3* [45] and *LAX3* is involved in AR formation (Fig. 1). Thus, it is most likely that *LBD29* also plays a role in AR formation.

We noticed that the complementation lines of the *lbd16* or *lbd18* mutant generated by expressing *LBD16* or *LBD18* under the control of their own promoter exhibited much higher expression levels of each transgene compared with that of the wild-type *LBD16* or *LBD18*, and yet the AR numbers in the complementation lines are comparable to that of the wild-type Col-0 [Fig. 3]. This result indicates that overexpression of a single *LBD* transcription factor gene regulated downstream of ARF7 is not sufficient to overproduce ARs in transgenic plants.

It has been previously reported that the apical part of *argonaute1* (*ago1*) mutants displays a defect in AR formation, but not in LR development, in response to auxin [31]. AGO1 is one of the components that plays a critical role in the regulation of posttranscriptional gene silencing [58]. *ARF17*, which is upregulated in *ago1* mutants, negatively regulates AR formation by repressing *GH3* genes and thus perturbing auxin homeostasis in a light-dependent manner [31]. Together, these results suggest that AR development has both unique and shared components with LR development in Arabidopsis. Identification of unique components, which play critical roles in AR development, aid in the discovery of the distinctive developmental processes between AR and LR development.

## Conclusions

The *ARF7*/*ARF19*-*LBD16*/*LBD18* transcriptional module via the AUX1/LAX3 auxin influx carriers plays an important role in AR formation in the Arabidopsis hypocotyl, suggesting that a common regulatory mechanism is utilized for LR and AR formation during auxin signaling.

## Methods

### Plant growth and tissue treatment

*Arabidopsis thaliana* seedlings were grown and treated as described previously [59]. For treatment with auxin IAA, seedlings were grown in a 16-h photoperiod on a 3 MM Whatman filter paper on top of agar plates at 23°C. The filter paper with seedlings was then transferred to a plate containing IAA at 20 μM and incubated for a given period of time with gentle shaking in the light at 23°C. The light intensity was approximately 120 μmol m^− 2^ s^− 1^ and was provided by three daylight wavelength color fluorescent bulbs (Kumho Electric Co.).

### Plant materials

The *Arabidopsis thaliana* ecotype Columbia (Col-0) was used in this study. We used the homozygous T-DNA insertion mutant lines *lbd16*, *lbd18*, *lax3, aux1–21, lbd16 lbd18, aux1 lax3, lax3 lbd16*, *lax3 lbd18*, *lax3 lbd16 lbd18*, *aux1 lbd16*, *aux1 lbd18*, *aux1 lbd16 lbd18*, *aux1 lax3 lbd16*, *aux1 lax3 lbd18*, and *aux1 lax3 lbd16 lbd18, arf7*, *arf19*, and *arf7 arf19*, which were developed in previous studies [37, 45, 51, 52]. We identified *arf6–1* (CS24606) and *arf8–2* (CS24608) knockout T-DNA insertion mutants from the Arabidopsis Biological Resource Center (ABRC). The *Pro*_*LBD16*_*:GUS* and *Pro*_*LBD18*_*:GUS* transgenic plants were obtained from a previous study [37]. *Pro*_*LAX3*_*:GUS* transgenic seeds were generously provided by Dr. Malcolm Bennett [51]. *Pro*_*ARF7*_*:GUS* (CS24633) and *Pro*_*ARF19*_*:GUS* (CS24634) transgenic seeds were obtained from the ABRC. To generate *Pro*_*LBD16*_*:LBD16:4xMyc* in the *lbd16* mutant background, the promoter region of *LBD16*, encompassing − 1309 to − 21 bp relative to the AUG codon, was amplified by PCR using the *pfu* DNA polymerase with primers harboring the *Not*I (N-terminus) and *Asc*I (C-terminus) sites, and was cloned into the pENTR™/SD/D-TOPO (Invitrogen) vector to yield *pENTR™/SD/D-TOPO:Pro*_*LBD16*_. The *LBD16* DNA fragment was inserted into the *pENTR™/SD/D-TOPO:Pro*_*LBD16*_ plasmid with the *Asc*I restriction site in both the N- and C-terminus to yield a *pENTR™/SD/D-TOPO:Pro*_*LBD16*_*:LBD16* plasmid. This construct was subcloned into the destination vector pGWB516 (Nakagawa, Shimane University, Japan) by an LR recombination reaction, and was then transformed into the *lbd16* mutant by Agrobacterium-mediated transformation, generating *Pro*_*LBD16*_*:LBD16:4xMyc*/*lbd16* Arabidopsis. *Pro*_*LBD18*_*:LBD18:3xHA*/*lbd18* Arabidopsis generated from a previous study was used [60]. *Pro*_*35S*_*:LBD16:GR*/*arf7 arf19* and *Pro*_*35S*_*:LBD18:GR*/*arf7 arf19* transgenic mutants were generated by crossing *arf7–1 arf19–1* (female) with *Pro*_*35S*_*:LBD16:GR* (male) or *Pro*_*35S*_*:LBD18:GR* (male) [37]. Homozygous lines were isolated according to genotype, the lack of lateral root phenotype for *arf7–1 arf19–1* and by PCR detection of genomic DNA for the *LBD16:GR* or *LBD18:GR* transgenes. All mutants and transgenic plants were confirmed via genotyping prior to usage. The primer sequences used in this study are shown in Additional file 2: Table S1.

### AR analysis

Induction of ARs in the hypocotyl was performed as previously described [32]. After seed sterilization, the seeds were sown on plates, incubated at 4°C for 2 d for stratification, and transferred to the light for several hours to induce germination. Plates were then placed vertically in the dark for 3 d, until the hypocotyls reached approximately 6 mm length, and were then transferred to the light for 7 d before counting the emerged ARs.

### RNA isolation and RT-qPCR analysis

Following treatment, Arabidopsis plants were immediately frozen in liquid nitrogen and stored at − 80°C. For the reverse transcription quantitative polymerase chain reaction (RT-qPCR) analysis, total RNA was extracted using an RNeasy Plant Mini Kit (Qiagen), and real-time RT-PCR was carried out using a QuantiTect SYBR Green RT-PCR kit (Qiagen) in a CFX96™ Real-time system using a C1000™ Thermal cycler (Bio-Rad) as described previously [61]. All RT-qPCR was conducted in triplicate biological replications and subjected to statistical analysis. Analysis of relative gene expression data for determining fold changes was conducted as described previously [60, 62]. Data analysis for determining the copy number of the transcripts and for determination of reaction specificities was performed as described previously [61]. RT-qPCR conditions and primer sequences are shown in Additional file 2: Table S1. The experimental conditions used for RT-qPCR followed MIQE (minimum information for publication of quantitative real time PCR experiments) requirements as described in Additional file 3: Table S2.

### Microscopy and histochemical GUS assays

Whole-mount visualization of the seedlings and histochemical assays for GUS activity were conducted as described previously [63].

### Statistical analysis

Quantitative data were subjected to statistical analysis for every pair-wise comparison using software for Student’s *t*-Test (Predictive Analytics Software for Windows version 20.0).

## Additional files

Additional file 1: **Figure S1.** Time-course expression of *LBD16* and *LBD18* in response to auxin in Col-0, *arf6* and *arf8* mutants. (PDF 110 kb)

Additional file 2: **Table S1.** Primer sequences and PCR conditions. (XLSX 12 kb)

Additional file 3: **Table S2.** Experimental conditions used in RT-qPCR based on MIQE requirements. (XLSX 10 kb)

## Abbreviations

AR: Adventitious root
ARF: Auxin response factor
Aux/IAA: Auxin/indole acetic acid protein
GUS: ß-glucuronidase
LBD/ASL: Lateral organ boundaries domain/asymmetric leaves2-like
RT-qPCR: Quantitative reverse transcription-PCR
