# Supplementary material for: LBD16 and LBD18 acting downstream of ARF7 and ARF19 are involved in adventitious root formation in Arabidopsis
Source: BMC Plant Biol. 2019 Jan 31;19:46. doi: 10.1186/s12870-019-1659-4 (PMC6357364; doi:10.1186/s12870-019-1659-4)
Supplement: Supplementary file 1 — Figure S1. Time-course expression of LBD16 and LBD18 in response to auxin in Col-0, arf6 and arf8 mutants. (PDF 110 kb) [file 12870_2019_1659_MOESM1_ESM.pdf]

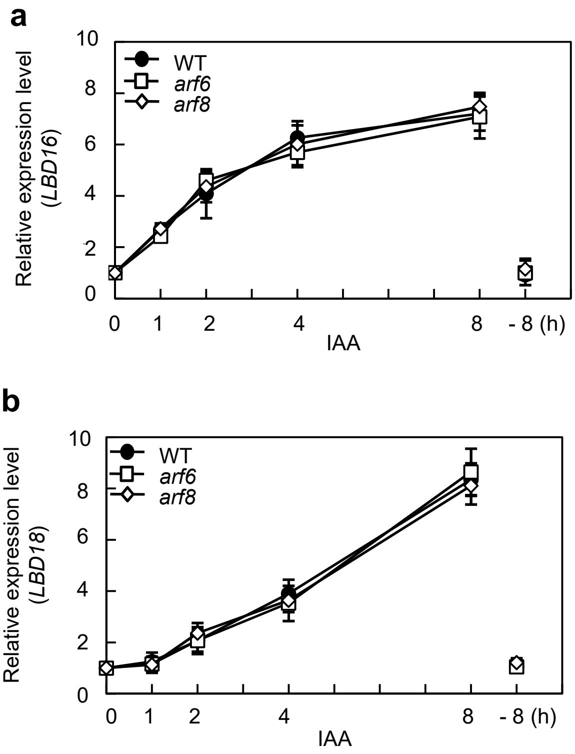

Supplementary Fig. S1 Time-course expression of *LBD16* and *LBD18* in response to auxin in Col-0, *arf6* and *arf8* mutants. (**a**) and (**b**) Expression analysis of *LBD16* (a) and *LBD18* (b) in Col-0, *arf6* and *arf8* in the presence of IAA at different time points. Seven-d-old seedlings were treated with or without 20 μM IAA for the indicated times, and the seedlings were harvested for RT-qPCR analysis. (–8 h indicates samples incubated with mock for 8 h). The relative fold changes were plotted after normalization to *ACTIN7*, and represent the ratio of the transcript level in the given treatment relative to the transcript level at time = 0. Mean ± SE values were determined from three biological replicates (each biological replicate was estimated as the average of two technical RT-qPCR replicates).
